# Avoidant-restrictive food intake disorder in a male patient with Goldenhar syndrome

**DOI:** 10.1007/s40519-022-01497-1

**Published:** 2022-10-30

**Authors:** Luca Bergonzini, Jacopo Pruccoli, Antonia Parmeggiani

**Affiliations:** 1 IRCCS Istituto Delle Scienze Neurologiche Di Bologna, Centro Regionale Per I Disturbi Della Nutrizione E Dell’Alimentazione in Età Evolutiva, U.O. Neuropsichiatria Dell’età Pediatrica, Bologna, Italy; 2 Dipartimento Di Scienze Mediche E Chirurgiche (DIMEC), Università Di Bologna, Bologna, Italy

**Keywords:** Goldenhar syndrome, Avoidant/restrictive food intake disorder, Children and adolescents, Pediatric

## Abstract

**Background:**

Goldenhar syndrome (GS) is a rare congenital condition characterized by the underdevelopment of structures deriving from the first and second branchial arches. Clinical phenotype might encompass extra-craniofacial abnormalities, and patients may experience neuropsychiatric disorders with a higher prevalence than healthy controls. To the best of our knowledge, an association between GS and Feeding and Eating Disorders (FED) has never been reported in the literature.

**Case report:**

A 15-year-old boy with GS was referred to our outpatient clinic due to severe underweight (BMI of 12.7 kg/m^2^) and food intake disorder with avoidant restrictive features. After a diagnosis of avoidant-restrictive food intake disorder (ARFID) was made, an inpatient multidisciplinary intervention and outpatient follow-up program were provided, which resulted in the improvement of the boy’s weight and FED psychopathology.

**Conclusions:**

The current report describes the first case of a young male with GS and ARFID. We suggest that ARFID may present itself as part of the spectrum of neuropsychiatric disorders associated with the syndrome; since traumatic experiences and gastrointestinal discomfort play a pivotal role in the development of ARFID among children, attention should be paid to those affected by GS that involves crucial structures in the swallowing process. Further literature evidence will help portray the complex relationship between ARFID and GS more precisely.

**Level of evidence:**

Level V, case report.

## Introduction

Goldenhar syndrome (GS) is a rare congenital condition characterized by the underdevelopment of structures deriving from the first and second branchial arches, such as masticatory muscles, mandible, maxilla, temporomandibular joint, facial nerves, ears, lip, tongue, palate, and teeth [1]. Extra-craniofacial anomalies have been documented in 35–55% of patients with GS and are mostly observed in the skeletal, circulatory, urogenital, and gastrointestinal tract [1, 2]. The spectrum of GS abnormalities ranges from mild to severe ones and includes patients with mild facial asymmetry to prominent facial defects, with more or less severe abnormalities of internal organs and/or skeleton [3].

Studies estimate that GS occurs in a wide incidence range, from 1:3500 to 1:45,000 live births, thus representing the second most common facial birth defect after cleft lip and palate [1, 3].

Overall, GS may cause aesthetic issues and several functional problems in patients, such as hearing impairment, obstructive sleep apnea (OSA), or feeding difficulty (FD), especially due to impaired suckling and chewing, incoordination of deglutition, and dysphagia [1]. Moreover, patients with GS may experience several neuropsychiatric disorders with a higher prevalence than healthy controls, such as cranial nerve palsies, intellectual disability, cognitive impairment, or autism spectrum disorder (ASD) [4, 5].

Interestingly, the association of GS with Feeding and Eating Disorders (FED) has never been reported in the literature, to the best of our knowledge. In the current report, we present the case of a young male with GS experiencing severe avoidant-restrictive food intake disorder (ARFID) symptoms with failure to thrive.

## Case report

A 15-year-old male was referred to a third-level Regional Center for Feeding and Eating disorders in developmental age due to severe underweight and a 2-year history of food intake disorder with avoidant restrictive features.

Familial history was positive for non-specified infantile epilepsy and intellectual disability in his father; his mother and his elder brother had no relevant medical history.

A diagnosis of GS was provided at birth. The patient’s phenotype comprises mild left hemifacial microsomia, left aural atresia, aplasia of the external auditory canal, and severe conductive hearing loss (with a 75 dB left auditory threshold). Global developmental delay (particularly affecting language production) and mild intellectual disability (IQ 53) were also reported, with motor clumsiness, toe walking, coordination, and balance impairment. Orthodontists and otolaryngologists highlighted a high-arched palate, class 2 malocclusion, rhinophony, and mild verbal and oral dyspraxia with visceral swallowing, which improved after a speech and myofunctional therapy. No cardiovascular involvement was documented at echocardiography and electrocardiography (ECG).

Ear computed tomography (CT) and high-resolution computed tomography (HRCT) showed medium and outer ear’s abnormal development, with dysmorphic ossicular chain and severely hypoplastic tympanic cavity, but normal inner ear and temporomandibular joint; involvement of VII and VIII cranial nerves was specifically ruled out. Brain and spine magnetic resonance imaging (MRI) was normal. Genetic evaluation with array-CGH did not yield diagnostic results. Lumbar dextroscoliosis resulted from a spine x-ray.

Feeding issues first occurred at the age of 13, when the patient started to gradually limit his food intake to pasta, tomatoes, apples, pears, and bananas, drinking only fruit juices, tea, or sparkling drinks and almost no water. His relatives described him as a picky eater since childhood; height and weight were consistently at the 10th percentile for age and sex until the age of 13. A year after, during the first national lockdown due to the COVID-19 pandemic, the patient experienced sleep–wake inversion, with complete disruption of the eating routine, worsening of selectivity, and reduced interest in eating. Once the pandemic restrictions eased, he continued to be socially isolated, rarely leaving his bedroom and ultimately withdrawing from school.

**Fig. 1:**
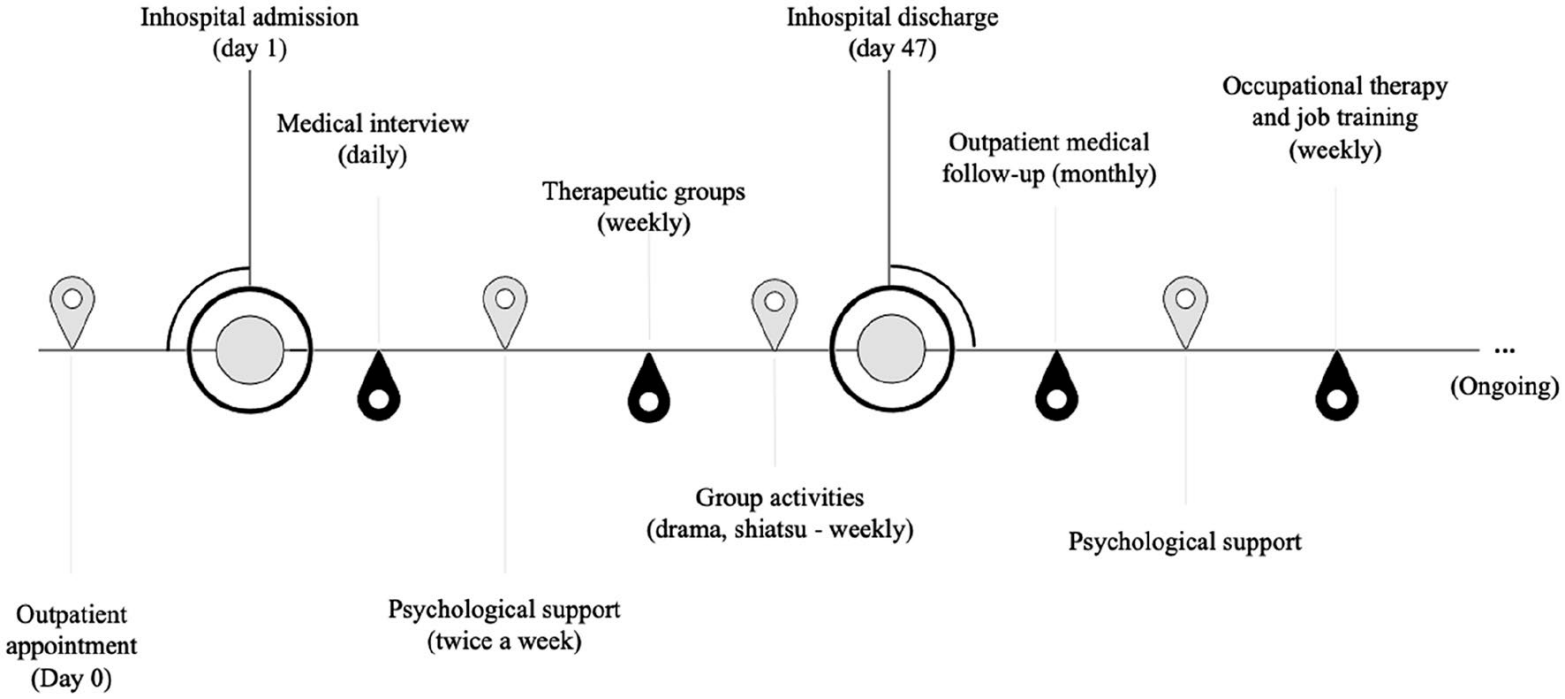
Inpatient and outpatient care and treatments timeline

When he first came to the Center, the patient was 15 years old; the physical examination highlighted GS aforementioned features, pale skin, disheveled appearance, slumped posture, and thin build. Height was 162 cm (7th percentile for age and sex); weight was 33.4 kg (under the 3rd percentile for age and sex); BMI was 12.7 kg/m^2^ (58% of median body mass index, under the 3rd percentile for age and sex). Absent fat mass resulted from bioelectrical impedance analysis. The neurologic exam highlighted muscle fatigue with no neurological deficits. During the mental status examination, he showed a cooperative attitude toward the examiner; however, eye contact was mainly avoidant, with several stereotyped activities that gradually diminished on closer acquaintance. He displayed an immature attitude with clingy features toward his mother. His mood was euthymic, with appropriate affect, normal speech volume, and clear and coherent features, but slight underproductivity. No hallucinations were highlighted and thought content appeared normal, with logical thought processes and normal perception. He showed distractibility during calculation tests (i.e., multiplication of two numbers) or working memory tasks (i.e., memorizing a list of written words). The patient showed no desire for weight loss, no eliminatory behaviors, and no fear regarding body weight or shape. He showed partial awareness about his condition and his low body weight, complaining about asthenia and pre-syncopal symptoms, although with absent insight about food selectivity. He was promptly hospitalized (Fig. 1) to receive medical help for his severe underweight. Blood tests and ECG were unremarkable, apart from high prolactin levels (28.9 ng/ml) and ALP levels (240 U/L). An average energy intake of 800–1000 kcal/day resulted from a food survey. He was supplied with a personalized nutritional program resulting from his own choice of daily meals with the supervision of a dietician, providing a daily energy intake of 1300 kcal/day up to 2100 kcal/day. Psychological support was provided twice a week (Fig. 1).

During his stay in the inpatient clinic, the patient showed a tendency to self-isolate and focused interests (he would stay in his room to play with tablets and videogames the whole day); therefore, he experienced several challenges in relating to hospitalized peers (5 girls aged 10–17 years old with anorexia nervosa or bulimia nervosa). He was also only partially compliant with the provided psychological support: he repeatedly displayed discomfort during psychological interviews, manifesting extreme difficulties with projective tests or psychometric testing tools, and was reluctant to take part in therapeutic groups and activities.

Despite these difficulties, several psychometric tests were administered, including the Eating Disorder in Youth Questionnaire (EDY-Q, Table 1) [6], the Nine Item Avoidant/Restrictive Food Intake disorder screen (NIAS, Table 2) [7], and the Autism Diagnostic Observation Schedule – Second edition (ADOS-2) [8]. With regards to EDY-Q and NIAS, items related to limited intake (food avoidance emotional disorder) and appetite subscale scored the highest. Neither EDY-Q nor NIAS highlighted significant scores in the field of fear subscale (aversive subtype/functional dysphagia) and peaky eating subscale (limited variety subtype/selective eating). Weight problems (items 4 and 5) and distorted cognitions about weight and shape (items 6 and 7) were reported at least “often” and less than “sometimes”, respectively, at the EDY-Q. Assessment of communication and reciprocal social interaction through ADOS-2 was suggestive of ASD.

**Table 1:** EDY-Q [6]

EDY-Q concept	Item	Score at hospital admission	Score at six months follow-up
Limited intake subtype (Food avoidance emotional disorder)	Food avoidance	5/6	1/6
	Lack of interest in food	4/6	5/6
	Emotional food avoidance	1/6	1/6
Limited variety subtype (selective eating)	Selective eating behavior	1/6	2/6
	Avoidance to try new foods	5/6	1/6
	Sensory food avoidance	1/6	0/6
Aversive subtype (functional dysphagia)	Fear of choking	1/6	0/6
	Fear of swallowing	1/6	0/6
Weight problems	Underweight	5/6	1/6
	Wish to gain weight	5/6	5/6
Weight and shape concern (distorted cognition)	Weight concern	0/6	0/6
	Shape concern	0/6	0/6

*EDY-Q* Eating Disorder in Youth Questionnaire. High scores among examples of ARFID subtypes and weight problems, with the absence of distorted cognition, gauge the presence of ARFID [6]

**Table 2:** NIAS Child [7] (scores at the time of hospital admission)

NIAS subscales	Score at hospital admission	Score at six months follow-up
Peaky eating subscale	5/15	6/15
Appetite subscale	10/15	5/15
Fear subscale	3/15	6/15

*NIAS* Nine item avoidant/restrictive food intake disorder screen. High scores are associated with positive screening for ARFID [7]

In light of the aforementioned data, a diagnosis of ARFID and ASD was made by two child neuropsychiatrists after a multidisciplinary evaluation by a team of physicians, psychologists, and dieticians. The diagnosis was provided according to the Diagnostic and Statistical Manual of Mental Disorders (DSM-5) criteria, as a result of the aforementioned psychometric evaluations and clinical interviews [9]. By the end of his stay, the patient became gradually compliant with the nutritional program; he reached a body mass index of 14.75 kg/m^2^. An outpatient multidisciplinary follow-up program was scheduled to provide clinical support (Fig. 1); 6 months after hospital discharge, the BMI was 15.3 kg/m^2^, and NIAS and EDY-Q showed improvement among limited intake-related scores and appetite subscale, respectively.

## Discussion

Organic feeding disturbances in GS have been documented in 26.4% of patients in a sample of 755 patients affected in a large multicenter study; risk factors for organic feeding disturbances include bilateral facial involvement, severe mandibular hypoplasia, cleft lip/palate, presence of OSA and extra-craniofacial anomalies [2]. The patient showed no clear-cut risk factors or evidence of organic feeding disturbances due to GS during hospitalization; no swallowing dysfunctions were highlighted in several evaluations; therefore, his severe condition of FED could not be directly ascribed to GS malformations. A diagnosis of ARFID with GS and ASD was considered. Patients with ARFID show persistent avoidance of certain foods and/or restriction of overall intake, which results in a diet limited in variety and/or volume unable to meet nutritional and/or energy needs [10]. These features constitute a psychiatric disorder when they are associated with one or more dysfunctional clinical features, such as significant weight loss (or failure to thrive), significant nutritional deficiency, dependence on enteral feeding or oral nutritional supplements, and marked interference with psychosocial functioning [9]. Three specific ARFID subtypes are described. First, “ARFID-limited intake” identifies weight loss and medical compromise as a consequence of apparent low appetite and lack of interest in eating; second, “ARFID-limited variety” concerns patients in which food avoidance and restriction are due to sensory sensitivity (“peaky eaters”); third, “ARFID-aversive” refers to patients experiencing fear of aversive consequences from eating, such as nausea, choking, gagging or pain [10]. In this case, the identification of a specific ARFID subtype was hampered by the diagnosis of mild intellectual disability, which made the assessment through psychometric scales challenging due to difficulties in focusing on a task and abstracting. Sensory sensitivity or interest in eating could not be reliably assessed; the ARFID-aversive subtype could not be ruled out since the patient’s history was positive for mild oral dyspraxia with visceral swallowing due to GS during childhood, which may have contributed to the development of feeding-related discomfort and food avoidance. Eventually, a diagnosis of ARFID with GS and ASD was confirmed, according to DSM-5 criteria [9] given the patient’s severe failure to thrive, which required multidisciplinary assistance from a tertiary care center for FED in developing age, both in an inpatient and outpatient setting.

One may ask whether avoidant-restrictive behavior could be due, in this case, to ASD. The final score resulting from the administration of ADOS-2 by a trained psychologist was consistent with autistic spectrum features and showed the patient’s impairment in reciprocal social interaction and communication, below what is normally expected for his general developmental level. Moreover, providing inpatient psychological support was challenging due to the patient’s highly focused interests and his tendency to self-isolate and avoid psychological interviews, therapeutic groups, and activities. The literature reports that ASD is more common among individuals with GS than in the general population, although the occurrence of intellectual disability and sensory impairments (i.e., deafness) in the syndrome may hinder the identification of the disorder in GS patients, leading to the over- or under-diagnosis of ASD [3, 5]. Besides, it is well known that the prevalence of atypical eating behaviors is higher among individuals with ASD than among children in the general population; indeed, children with ASD generally present rigid eating behaviors and heightened sensory sensitivity, with limited and brand-specific food preferences [9, 12]. However, these features do not always result in the level of impairment that would be required for a diagnosis of ARFID, and a thorough clinical assessment is crucial to differentiating feeding problems from disordered eating in ASD patients [11].

In conclusion, we suggest that ARFID may present itself as part of the spectrum of neuropsychiatric disorders associated with GS. In this case, it is still unclear whether the occurrence of ARFID could be either a consequence of previous oral dyspraxia and visceral swallowing due to GS or a result of arduous interoception about internal body states, such as hunger, due to ASD, or both. However, given the pivotal role of traumatic experiences (i.e., acute episodes of choking) and gastrointestinal discomfort in the development of ARFID among children, we suggest that particular attention should be paid to those infants and children affected by syndromes involving crucial structures for the complex and dynamic process of swallowing [10].

This case report highlights the fundamental role of specific screening for FED among patients with complex congenital syndromes, like GS, and other neuropsychiatric comorbidities, such as ASD. The patient’s improvement in weight and FED psychopathology during the outpatient follow-up program suggests that the development of multidisciplinary treatment protocols based on psychological, nutritional, and medical support would help to guarantee a timely and effective intervention in such complex clinical pictures. Further literature evidence will definitely help to precisely map the complex relationship between FED and GS.

## Strengths and limitations

The present case report describes for the first time the occurrence of severe Feeding and Eating Disorders in a young patient with Goldenhar syndrome. The clinical and psychometric assessment was limited by the patient’s mild intellectual disability.

## What is already known on this subject?

Patients with Goldenhar syndrome may experience neuropsychiatric disorders with a higher prevalence than healthy controls, such as cranial nerves palsies, intellectual disability, cognitive impairment, or autism spectrum disorder.

## What this study adds?

To the best of our knowledge, an association between Goldenhar syndrome and Feeding and Eating Disorders has never been reported in the literature. The present case report describes the occurrence of avoidant/restrictive food intake disorder in a patient with Goldenhar syndrome.
